# Similarities in transcription factor IIIC subunits that bind to the posterior regions of internal promoters for RNA polymerase III

**DOI:** 10.1186/1471-2148-4-26

**Published:** 2004-08-09

**Authors:** Sachiko Matsutani

**Affiliations:** 1 Division of Microbiology, National Institute of Health Sciences, Setagaya-ku, Tokyo 158-8501, Japan

## Abstract

**Background:**

In eukaryotes, RNA polymerase III (RNAP III) transcribes the genes for small RNAs like tRNAs, 5S rRNA, and several viral RNAs, and short interspersed repetitive elements (SINEs). The genes for these RNAs and SINEs have internal promoters that consist of two regions. These two regions are called the A and B blocks. The multisubunit transcription factor TFIIIC is required for transcription initiation of RNAP III; in transcription of tRNAs, the B-block binding subunit of TFIIIC recognizes a promoter. Although internal promoter sequences are conserved in eukaryotes, no evidence of homology between the B-block binding subunits of vertebrates and yeasts has been reported previously.

**Results:**

Here, I reported the results of PSI-BLAST searches using the B-block binding subunits of human and *Shizosacchromyces pombe *as queries, showing that the same *Arabidopsis *proteins were hit with low *E*-values in both searches. Comparison of the convergent iterative alignments obtained by these PSI-BLAST searches revealed that the vertebrate, yeast, and *Arabidopsis *proteins have similarities in their N-terminal one-third regions. In these regions, there were three domains with conserved sequence similarities, one located in the N-terminal end region. The N-terminal end region of the B-block binding subunit of *Saccharomyces cerevisiae *is tentatively identified as a HMG box, which is the DNA binding motif. Although I compared the alignment of the N-terminal end regions of the B-block binding subunits, and their homologs, with that of the HMG boxes, it is not clear whether they are related.

**Conclusion:**

Molecular phylogenetic analyses using the small subunit rRNA and ubiquitous proteins like actin and α-tubulin, show that fungi are more closely related to animals than either is to plants. Interestingly, the results obtained in this study show that, with respect to the B-block binding subunits of TFIIICs, animals appear to be evolutionarily closer to plants than to fungi.

## Background

Phylogenetic relationships among animals, fungi, and plants have been a controversial issue. Although fungi traditionally had been considered more closely related to plants than to animals, Whittaker and Margulis [1] classified the fungi as a separate kingdom in their five-kingdom classification: the three major multicellular groups of animals, fungi, and green plants were each given the status of kingdoms derived from different protistan lineages of uncertain affinities. With the determination of the primary structures of homologous macromolecules in various organisms, spanning several kingdoms, molecular phylogenetic techniques resulted in new hypotheses about the relationships among eukaryotes. Small subunit rRNA, and the proteins like actin and α-tubulin, exist ubiquitously and their primary structures are highly conserved. Thus, these sequences have been used to make molecular trees [for examples, [2,3]]. Most of these studies place fungi as more closely related to animals than either is to plants [3-6].

Eukaryotic RNA polymerase III (RNAP III) transcribes a variety of small RNAs like tRNAs, 5S rRNA, and several viral RNAs [7]. Short interspersed repetitive elements (SINEs) are also transcribed by RNAP III [8]. Genes for these small RNAs have internal promoters that consist of two regions called the A and B blocks [9]. These promoter sequences are well-conserved in diverse eukaryotes [9]. Transcription by RNAP III requires the multisubunit transcription factor TFIIIC, which plays an important role in transcription initiation [10]. TFIIIC contains a B-block binding subunit, which recognizes the RNAP III promoter in the transcription of tRNAs and several viral RNAs, orienting its associated subunits along the DNA [11]. TFIIIC that is oriented toward the start site, promotes TFIIIB binding and assists in directing accurate initiation by RNAP III [12,13].

In TFIIIC of *Saccharomyces cerevisiae*, a subunit of 138 kDa binds to a B-block, and its gene which is called *TFC3*, has been cloned [14]. The open reading frame for the B-block binding subunit is interrupted by one intron [14]. *TFC3 *is a single-copy gene and essential for cell viability [14]. In human and rat, the B-block binding subunits of TFIIICs are 243 kDa and 220 kDa respectively [14,15], and there is great similarity between them at the amino acid sequence level [15]. However, B-block binding subunit from mammals has been thought to show no homology to the *S. cerevisiae *138 kDa subunit, although all of them bind to similar DNA regions, suggesting a significant degree of evolutionary divergence for RNAP III factors [15,16]. Huang et al. [17] identified several subunits of *S. pombe *TFIIIC from the *S. pombe *sequence database by homology searches using the *S. cerevisiae *TFIIIC subunits as queries; one of these subunits named Sfc3p, is similar to the *S. cerevisiae *B-block binding subunit. It has been thought that, like the B-block binding subunit from *S. cerevisiae*, Sfc3p does not share homology with the human B-block binding subunit. On the other hand, Sfc1p, Sfc4p, and Sfc6p, which are other subunits of *S. pombe *TFIIIC, show homologies not only to *S. cerevisiae *TFIIIC subunits but also to human TFIIIC subunits [17]. It has been shown that Sfc1p, Sfc3p, Sfc4p, and Sfc6p are associated *in vivo, *and the isolated Sfc3p complex is active in the *in vitro *RNAP III-mediated transcription of *S. pombe *tRNA genes [17].

**Figure 1:**
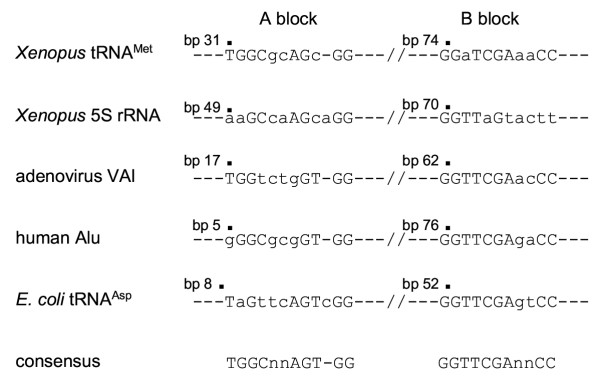
**Sequences of the internal promoters for RNA polymerase III. **In the sequences of the *Xenopus *tRNA^Met ^gene [37], the *Xenopus *5S rRNA gene [38], adenovirus VAI [39], human Alu [40], and the *E. coli *tRNA^Asp ^gene [18], the nucleotides identical to those in the consensus sequence [9] are shown by capital letters.

The lack of clear homology between the yeast and human B-block binding subunits is strange, because transcription of tRNA genes by RNAP III is initiated by the binding of these subunits to the B-block regions, and the internal promoter sequences are highly conserved between vertebrates and yeasts (see above). Interestingly, bacterial tRNA genes also have conserved RNAP III promoters (Fig. 1) [18]. Although these genes are transcribed from the upstream promoters by the bacterial RNA polymerase, RNAP III can transcribe some of them *in vitro *[18]. Thus, understanding the relationship of the B-block binding subunits from human and yeast TFIIICs is important for understanding the evolution of the RNAP III transcription machinery. Here, I demonstrate that, at the amino acid sequence level, the B-block binding subunits of the vertebrate and yeast TFIIICs do have important homologies. These homologies are found by comparing the B-block binding subunits with *Arabidopsis *proteins, which appear to be the homologs of both the human and yeast subunits.

## Results

### PSI-BLAST search using the human B-block binding subunit as a query

**Figure 2:**
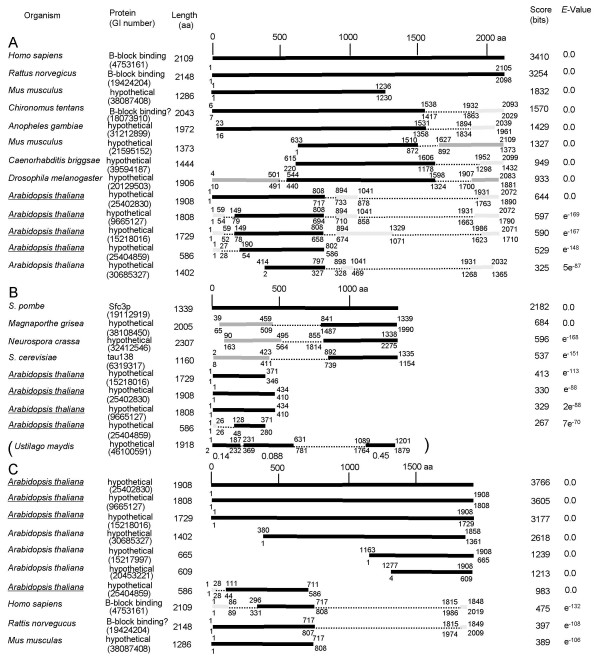
**Proteins which show homologies to the B-block binding subunits of human and *S. pombe*, and the *Arabidopsis *protein GI 25402830. A. **The result of a PSI-BLAST search using the human subunit (GI 4753161) as a query. **B. **The result of a PSI-BLAST search using the *S. pombe *subunit (GI 19112919) as a query. **C. **The result of a PSI-BLAST search using the *Arabidopsis *protein (GI 25402830) as a query. In A-C, only the proteins that had bit scores more than 200 are shown. Bold horizontal lines (black, dark gray, and pale gray lines) are the regions that appeared as convergent iterative alignments. The numbers above the lines are the amino acid positions in the query sequence, and those beneath the lines are the amino acid positions in the hit sequences. When more than two regions were hit in the same sequence, the highest bit score and *E*-value are shown. Black lines are the alignments that had the best bit scores and *E*-values. Gray-colored alignments had the worse scores. Dark gray lines represent the alignments with the bit scores more than 200, and the scores of the pale grey alignments are lower than 200. The proteins that had similarities only in short regions (smaller than about 300 amino acids) were omitted, even if the bit scores were more than 200. The case of the *Ustilago *sequence is exceptional. This result was from the PSI-BLAST which was limited a search of the fungi database. The bold horizontal lines are the regions that appeared as convergent iterative alignments, and below them *E*-values are shown.

When the B-block binding subunits of human and *S. cerevisiae *are aligned using the early Clustal program, they show little sequence similarity [16]. To search for potentially homologous B-block binding regions, first I carried out a PSI-BLAST search where the human subunit was used as a query. PSI-BLAST is known to be useful in finding distantly related proteins [19]. After three iterations, twelve sequences were found with strong similarities to the sequence of human B-block binding subunit. Fig. 2A shows a summary of the result of a PSI-BLAST search, including proteins with alignment scores better than 200. Proteins that showed only local alignment similarities (shorter than about 300 amino acids) were omitted. The human B-block binding subunit was very similar to the rat subunit (75% identity and 84 % positive) as reported by Lagna et al. [15] (Fig. 2A). The nuclear protein of 238 kDa in the mosquito *Chironomus tentans *(GenBank identification number: 18073910) is structurally similar to the human B-block binding subunit [20]; in BLAST and FASTA searches of non-redundant databases using *C. tentans *protein as a query, the best hits were the human and rat B-block binding subunits with 28 % identities. A comparable level of similarity has been found also in the *Drosophila *hypothetical protein, but no other related proteins were identified in the database [20]. Immunoelectron microscopy shows that this mosquito protein is located at sites of transcription, suggesting the role of the protein in transcription initiation [20]. In this study, when a PSI-BLAST search was performed using the human sequence as a query, the *Chironomus *and *Drosophila *proteins were hit (23 % and 20 % identities respectively) (Fig. 2A). In addition to these, hypothetical proteins from the mosquito *Anopheles gambiae *(GI 31212899), *Mus musculus *(GIs 38087408, and 21595152), *Caenorhabditis briggsae *(GI 39594187), and *Arabidopsis thaliana *(GIs 25402830, 9665127, 15218016, 25404859, and 30685327), were also hit with low *E*-values: *E*-values were 0, 0, 0, 0, 0, e^-169^, e^-167^, e^-148^, and 5e^-87^, respectively (Fig. 2A). Interestingly, in *Arabidopsis *proteins of GIs 25402830, 9665127, and 15218016, both the N- and C-terminal end regions corresponded to those of the human subunit, suggesting that these *Arabidopsis *proteins are orthologs of the B-block binding subunits (Fig. 2A).

### PSI-BLAST using the yeast B-block binding subunits as queries

The *S. cerevisiae *B-block binding subunit exhibits 21 % identity and 39 % similarity to the *S. pombe *Sfc3p protein, and these similarities extend to the overall sequences (Background; [17]). When I performed a PSI-BLAST search using the *S. cerevisiae *B-block binding subunit as a query, five sequences were hit with *E*-values better than threshold after three iterations: the *Neurospora crassa *hypothetical protein (GI 32412546) with 0.0, *S. pombe *Sfc3p with e^-159^, the *Magnaporthe grisea *hypothetical protein (GI 38108450) with e^-121^, the *Saccharomyces bayanus *hypothetical protein fragment (GI 10863079) with e^-100^, and the *Arabidopsis *hypothetical protein (GI 15218016) with 0.002 (data not shown). It is noteworthy that the *Arabidopsis *protein of GI 15218016 was hit although the *E*-value was not so good. This protein was hit also in the PSI-BLAST search using the human B-block binding subunit as a query (Fig. 2A).

Next, I performed a PSI-BLAST search using the *S. pombe *Sfc3p protein as a query. Fig. 2B is a summary of the result. The *Magnaporthe grisea *and *Neurospora crassa *hypothetical proteins (GI 38108450 and GI 32412546), were hit with very good *E*-values (0 and e^-168 ^respectively) (Fig. 2B). The *Aspergillus nidulans *protein GI 49107000 also was hit with a robust *E*-value (data not shown). Four hypothetical proteins of *Arabidopsis thaliana *were hit with *E*-values worse than those of the two fungi proteins but well above the threshold: GI 15218016 with e^-113^, GI 25402830 with e^-88^, GI 9665127 with 2e^-88^, and GI 25404859 with 7e^-70 ^(Fig. 2B). The *Arabidopsis *protein GI 15218016 was found also in the result of the PSI-BLAST search using the *S. cerevisiae *subunit as a query, but in the search with *S. pombe *Sfc3p it had a much better *E*-value. Surprisingly, these four *Arabidopsis *proteins were identical to the proteins that were hit with low *E*-values in the PSI-BLAST search using the human B-block binding subunit as a query (Fig. 2A). While both the N-terminal half regions and C- terminal end regions were similar between the *Arabidopsis *proteins and the human subunit, their similarities to *S. pombe *Sfc3p were only in the N-terminal halves (Figs. 2A and 2B). The B-block binding subunits of rat and human also were hit in this search, but with *E*-values of 5e^-5 ^and 0.20, respectively (data not shown); the N-terminal 350 amino acid sequences of the rat and human subunits showed similarities to the N-terminal region of *S. pombe *Sfc3p protein.

### PSI-BLAST using the B-block binding subunit homolog found in Arabidopsis as a query

The four *Arabidopsis *proteins (GIs 25402830, 9665127, 15218016, and 25404859), were hit with low *E*-values in both of the PSI-BLAST searches using the human and *S. pombe *subunits as queries. Thus, I performed a PSI-BLAST search using one of these *Arabidopsis *proteins (GI 25402830) as a query. Fig. 2C shows a summary of the result. Six hypothetical *Arabidopsis *proteins were hit with *E*-values of 0, and three of them were identical to the proteins which were hit in the PSI-BLAST searches using the human and *S. pombe *subunits as queries. In addition to these, the human and rat subunits were hit with low *E*-values (e^-132 ^and e^-108 ^respectively). The N-terminal 700 amino acids of the human and rat subunits were most similar to the *Arabidopsis *proteins, but the short regions of the C-terminal ends also were similar (Fig. 2C). The hypothetical mouse protein (GI 38087408) which was hit in the PSI-BLAST search using the human subunit as a query, also was hit in the PSI-BLAST search with *Arabidopsis *GI 25402830 (Fig. 2C). The B-block binding subunits of *S. pombe *and *S. cerevisiae *were hit with *E*-values worse than threshold (0.024 and 1.1 respectively) (data not shown). However, it should be noted that these *Arabidopsis *proteins were hit with *E*-values better than threshold when a PSI-BLAST search was performed using the *S. pombe *B-block binding protein as a query.

It is interesting that the four *Arabidopsis *proteins were similar to the B-block binding subunits of vertebrates and yeasts, despite the fact that vertebrate and yeast subunits share no recognizable homology [15-17]. These results seemed to imply that the *Arabidopsis *proteins of GIs 25402830, 9665127, 15218016, and 25404859 represent a 'missing link' between the vertebrate and yeast B-block binding subunits.

### Alignment of the B-block binding subunits and their homologs

**Figure 3:**
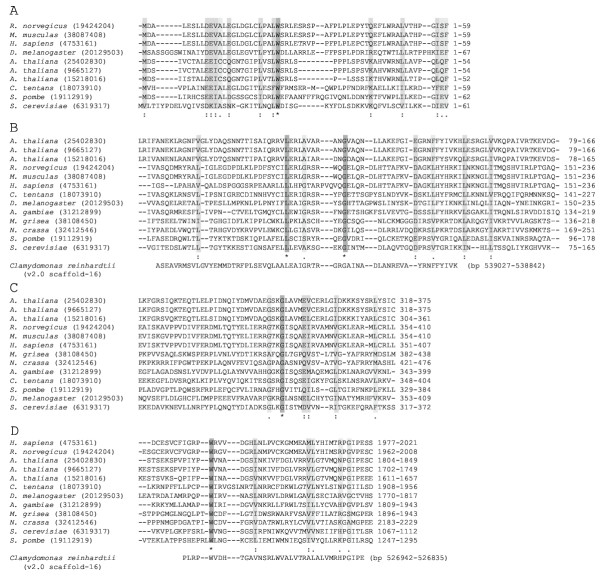
**Clustal W alignments of the sequences conserved in the B-block binding subunits and their homologs. **The GI numbers of the proteins are shown in parentheses. The amino acid positions of the sequences are shown to the right. The cases of the *Clamydomonas *sequences are exceptional. These sequences were hit by tblastn of the *C. reinhardtii *genome sequence using the *Arabidopsis *protein GI 25402830 as a query (see also Fig. 5). **A. **Alignment of the N-terminal end sequences. **B and C. **Alignments of the internal sequences of the proteins. **D. **Alignment of the C-terminal end sequences.

When the primary structures of human and rat B-block binding subunits and their homolog in *Drosophila *are compared, the most conserved sequences are located within the N-terminal two-thirds of the proteins, while the C-terminal one-third is much less conserved [20]. The *Chironomus tentans *protein, which probably binds to the B-block in the RNAP III promoter, also has similarities to the human, rat, and *Drosophila *proteins in the N-terminal region [20]. The results of PSI-BLAST searches here also showed that conserved sequences in the human subunit, and the three *Arabidopsis *homologs (GIs 25402830, 9665127, and 15218016), mainly are located in the N-terminal halves of the proteins (Fig. 2A). Similarly, conserved sequences in the *S. pombe *subunit and the three *Arabidopsis *homologs (GIs 25402830, 9665127, and 15218016), are located in the N-terminal one-third regions of the proteins (Fig. 2C). Thus, I used the Clustal W program [21] to align the N-terminal one-third regions of the proteins of human, rat, mouse, *Drosophila*, *Chironomus*, *Arabidopsis*, and yeasts. The Clustal W alignment of the N-terminal ends of about 60 amino acids corresponded to each of the alignments obtained by the PSI-BLAST searches between the query and hit sequences (Fig. 3A). However, the rest of the Clustal W alignment did not correspond to the alignments obtained by PSI-BLAST (data not shown). Therefore, I compared all of the PSI-BLAST alignments further by eye. Three regions including the N-terminal ends were found to be most conserved between the PSI-BLAST alignments; these three regions were aligned separately by Clustal W (Fig. 3). Previously, Rozenfeld and Thuriaux [22] performed PSI-BLAST using the *S. cerevisiae *B-block binding subunit as a query. They identified two domains of about 70 amino acids, which were conserved in *S. cerevisiae *subunit and *Arabidopsis thaliana *protein of 1808 amino acids [22]: amino acid (aa) positions 333–361 of the *S. cerevisiae *subunit correspond to positions 334–362 of the *A. thaliana *protein, and positions 1079–1111 in *S. cerevisiae *correspond to positions 1716–1748 in *A. thaliana*. These local homologies are detected also in the human B-block binding subunit by visual inspection (aa positions 367–397 and 1987–2019) (Fig. 2 in [22]). The *Arabidopsis *protein reported in Rozenfeld and Thuriaux [22] appears to be the GI 9665127 protein hit in the PSI-BLAST searches here, because the lengths of their amino acid sequences are the same and the partial sequences shown in their paper are identical to those in the GI 9665127 protein. The alignment shown in Fig. 3C contains the domain reported by Rozenfeld and Thuriaux [22], and the sequences in the alignment shown in their paper are identical to those in this study.

When a PSI-BLAST search was performed using the human B-block binding subunit as a query, it was shown that the C-terminal region also is conserved (Fig. 2A); for examples, *Chironomus*, *Anopheres*, *Drosophila*, and *Arabidopsis *(GIs, 25402830, 9665127, and 15218016) proteins are hit with *E*-values of e^-29^, 3e^-7^, 6e^-54^, 2e^-20^, 2e^-39^, and 2e^-21 ^respectively (data not shown). These regions contain the domains shown to have sequence similarities [22] (see above). When a PSI-BLAST search was performed using the *S. pombe *B-block binding subunit as a query, alignments consisting of its C-terminal region and each of the *Magnaporthe*, *Neurospora*, and *S. cerevisiae *sequences were generated, but no homology to the C-terminal regions of the *Arabidopsis *proteins was suggested (Fig. 2B). However, in agreement with the result of Rozenfeld and Thuriaux [22], when the *S. cerevisiae *B-block binding subunit was used as a query, the C-terminal region of the *Arabidopsis *protein (GI 9665127) was hit after four iterations; aa positions 1032–1146 of the *S. cerevisiae *subunit aligned to positions 1673–1774 of the *Arabidopsis *protein (GI 9665127) with an *E*-value of 5e^-21 ^(data not shown). Consequently, the C-terminal sequences of the human, rat, mosquitoes, *Drosophila*, *Arabidopsis *and fungi proteins, can be aligned by Clustal W (Fig. 3D).

### Are the HMG boxes in the B-block binding proteins?

**Figure 4:**
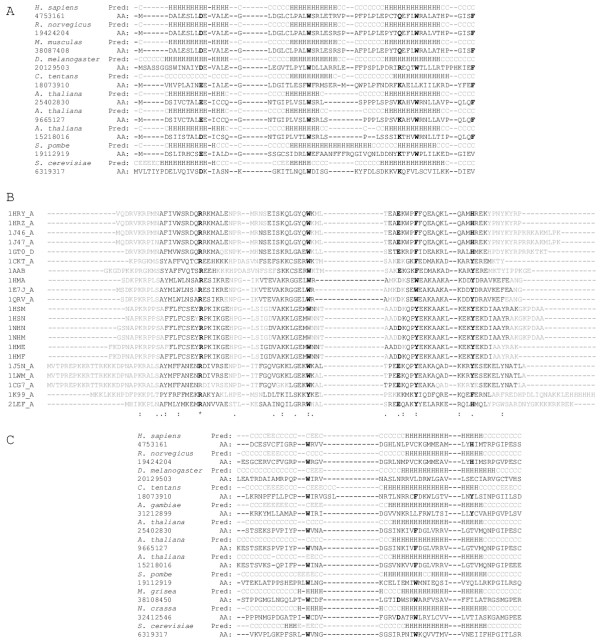
**Alignments of the N- and C- terminal end sequences of the B-block binding proteins, which are compared with the HMG box sequences. **Gaps were inserted into the sequences by visual inspection to relate the positions of the conserved basic and aromatic residues and the locations of the α-helices between the alignments. The amino acid residues common among the three alignments are shown in boldface. **A. **Alignment of the N-terminal end sequences. Above the amino acid sequences, are the secondary structures predicted from them, where H, E, and C represent helix, strand, and coil respectively. **B. **Alignment of HMG boxes. The identification codes for PDB entries  are shown to the left. The regions shown by black characters form α-helices (see ). **C. **Alignment of the C-terminal ends, with the predicted secondary structures shown above the amino acid sequences.

The aa positions 1–68 and 1037–1110 of the *S. cerevisiae *B-block binding subunit had been tentatively identified as HMG boxes [14]. The HMG box is a small eukaryotic DNA binding motif (70–80 amino acids in size) found in many proteins including transcription factors [23]. Interestingly, the regions of HMG boxes predicted for the *S. cerevisiae *subunit overlap the N- and C-terminal regions conserved in many B-block binding proteins. Therefore, I investigated whether these conserved regions could be homologs of HMG boxes. HMG boxes are diverse, and it is sometimes difficult to determine whether a given protein belongs to the HMG box superfamily [24]. However, in alignments of known HMG boxes a loose consensus sequence can be defined, in which many basic and aromatic residues are conserved [24-26] (see also Fig. 4B). Structures of several HMG boxes have been determined by the NMR spectroscopy and X-ray diffraction [for examples, [25,27,28]] (Fig. 4B); most of them have three α-helices arranged in L-shapes, and tertiary structures stabilized by conserved aromatic residues. It should be noted that in alignments of the N- and C-terminal end regions of B-block binding proteins, several basic and aromatic residues also are conserved (Figs. 3A and 3D). Since HMG boxes contain three helices (see above), I predicted the secondary structures of N- and C-terminal regions of B-block binding proteins using the PSIPRED method [29]. The results are shown in Figs. 4A and 4C. In the N-terminal end regions, all of the sequences (except for *Drosophila*) were predicted to contain three α-helices with corresponding locations (Fig. 4A). Although the *Drosophila *N-terminal sequence was predicted to have only two helices, their locations corresponded to the middle and posterior helices in the other sequences (Fig. 4A). In the C-terminal end regions, all of the sequences were predicted to contain one helix in the same corresponding location (Fig. 4C). Fig. 4 shows alignments of B-block binding protein sequences and HMG boxes, arranged to show relationships between them. Numerous gaps were inserted into the sequences by visual inspection in order to relate the positions of basic and aromatic residues and locations of the α-helices (Fig. 4). It appears possible that HMG boxes are present in B-block binding proteins, particularly in their N-terminal regions, however, strong evidence for this relationship is not clear from comparing their amino acid sequences.

### Evolutionary relationships of the B-block binding proteins

**Figure 5:**
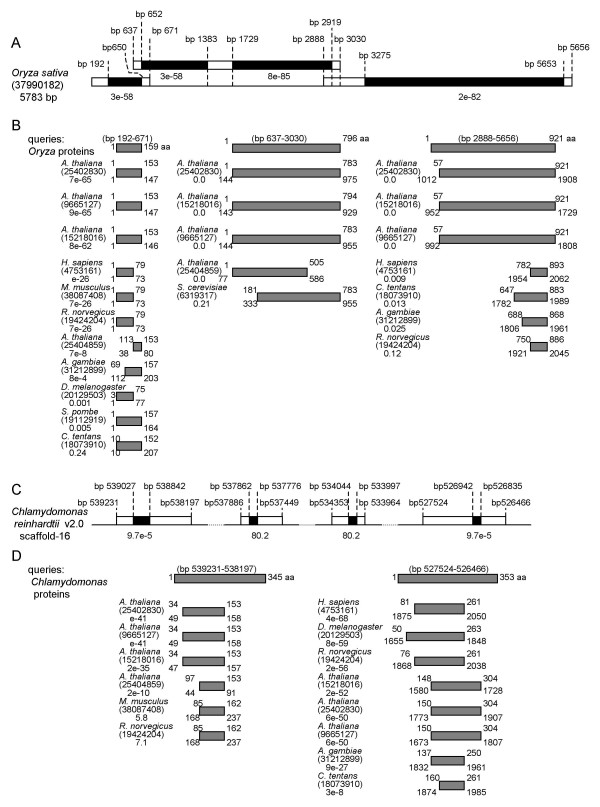
**Protein coding regions in the nucleotide sequences of *Oryza sativa *and *Chlamydomonas reinhardtii *with predicted amino acid sequences similar to B-block binding proteins. A. **Protein coding regions in the *Oryza sativa *nucleotide sequence GI 37990182 with amino acid sequences similar to the *Arabidopsis *protein GI 25402830. Coding regions are shown as boxes and the regions hit by a tblastn search are the filled boxes. *E*-values are shown below the filled boxes. **B. **The B-block binding proteins hit by PSI-BLAST searches using the *Oryza *three amino acid sequences as queries. The corresponding bp positions are indicated above the queries, and hit sequences are below the *Oryza *queries. Only proteins with *E*-values better than 10 are shown. **C. **Protein coding regions in the *Chlamydomonas reinhardtii *nucleotide sequence with amino acid sequences similar to the *Arabidopsis *protein GI 25402830. Coding regions are shown as boxes, and the regions hit by a tblastn search are shown as filled boxes. *E*-values are shown below the filled boxes. **D. **The B-block binding proteins which were hit by PSI-BLAST searches using the *Chlamydomonas *amino acid sequences as queries. The corresponding bp positions are indicated above the queries, and hit sequences are below the *Chlamydomonas *queries. Only proteins with *E*-values better than 10, are shown.

PSI-BLAST searches presented here provide the evidence that the B-block binding subunits of vertebrates and yeasts are homologous, and that the *Arabidopsis *proteins can be used to link these subunits. These results suggest that, with respect to the B-block binding subunits of TFIIICs, animals are evolutionarily closer to *Arabidopsis *than to yeasts. These results are intriguing because phylogenetic analyses using sequences of small subunit rRNA, elongation factor 1, actin, α-tublin, β-tubulin, and heat shock protein 70, show that animals and fungi are most closely related, to the exclusion of the broad diversity of eukaryotic phyla including plants [3-6]. To confirm that the B-block binding subunits of animals and plants are closely related, I decided to carry out an extensive comparison of the sequences from additional plant taxa. However, the PSI-BLAST searches using the B-block binding subunits of human and *S. pombe*, and *Arabidopsis *homolog (GI 25402830) as queries, did not significantly hit any of the plant sequences except the *Arabidopsis *proteins shown in this study. Therefore, I performed a tblastn search of the Viridiplantae database using the *Arabidopsis *protein GI 25402830 as a query. The *Oryza sativa *sequence GI 37990182 was hit with the best *E*-value (8e^-85^) among Viridiplantae sequences, except the *Arabidopsis *genes already described (Fig. 5A). In this *Oryza *sequence, there are four regions that show high similarities to the query *Arabidopsis *protein (Fig. 5A). This result suggests that the *Oryza *sequence GI 37990182 encodes a B-block binding protein. Subsequently, I performed the PSI-BLAST searches using three conserved domains from the *Oryza *sequence of GI 37990182 (Fig. 5B). The query sequence from the 5' end of the *Oryza *gene had greater similarities to the B-block binding proteins in animals than those in fungi: for examples, the human and rat subunits were hit with *E*-values of e^-26 ^and 7e^-26 ^respectively, while the *S. pombe *subunit was hit with an *E*-value of 0.005, and other fungal sequences were not hit with *E*-values better than 10 (Fig. 5B). The results for the query sequence from 3' region of the *Oryza *gene also indicate that the animal and plant proteins are more closely related: for examples, the human and rat subunits were hit with *E*-values of 0.009 and 0.12 respectively, while the yeast subunits were not hit with *E*-values better than 10 (Fig. 5B). The result for the query sequence from the middle region of the *Oryza *gene, however, did not retrieve animal subunits, although the *S. cerevisiae *subunit was hit with an *E*-value of 0.21 (Fig. 5B).

I also searched for a B-block binding protein homolog in the genome of green alga *Chlamydomonas reinhardtii*. I performed a tblastn search using the *Arabidopsis *protein GI 25402830 as a query and the *C. reinhardtii *genome sequence ver2 in the Joint Genome Institute website (see Methods). The *Arabidopsis *sequences at aa positions of 83–144, 234–262, 698–713, and 1812–1847 showed similarities to sequences corresponding to bp positions of 539027-538842, 527862-537776, 534044-533997, and 526942-526835 in the *C. reinhardtii *scaffold 16, with *E*-values of 9.7e^-5^, 80.2, 80.2, and 9.7e^-5 ^respectively (Fig. 5C). The amino acid sequences deduced from bp positions 539027-538842 and 526942-526835 corresponded to the domains conserved among the B-block binding proteins that were aligned by Clustal W, as shown in Figs. 3B and 3D. These results suggest that the *Chlamydomonas *B-block binding protein is encoded in these DNA regions. Subsequently, I performed PSI-BLAST searches using the two amino acid sequences of *C. reinhardtii *with the highest similarities to the *Arabidopsis *protein (Fig. 5D). The query sequence from bp positions 539231-538197 in *C. reinhardtii*, showed similarities to the rat B-block binding subunit and its homolog in mouse (*E*-values of 7.1 and 5.8 respectively) (Fig. 5D). Although these *E*-values are not robust, no fungal B-block binding proteins was hit with *E*-values better than 10. The other query sequence encoded in bp positions 527524-526466 of *C. reinhardtii *also had greater similarities to the B-block binding proteins in animals than to those in fungi: for examples, the human and rat subunits were hit with *E*-values of 4e^-68 ^and 2e^-56 ^respectively, while no fungal proteins were hit with *E*-values better than 10 (Fig. 5D). The results of these PSI-BLAST searches with *Oryza *and *Chlamydomonas *query sequences indicate that the greater similarity in TFIIIC B-block binding proteins between animals and plants, with yeast as more distant, across the broad diversity of the animal and plant kingdoms.

Because yeasts may not be representative of all fungi, it is important to demonstrate that the greater similarity between the animal and plant B-block binding proteins extends beyond the yeast taxa. To this end, I searched for homologs of the B-block binding protein in the basidiomycete genomes. I performed a PSI-BLAST search using the *S. pombe *subunit as a query, limiting the search to the fungi database. The sequence hit with the best *E*-value among the basidiomycete sequences, was the *Ustilago maydis *protein GI 461005911 (Fig. 2B). The *Cryptococcus *and *Coccidiodes *sequences were not hit with *E*-values better than 10. In the *Ustilago *sequence GI 461005911, three regions show similarities to the *S. pombe *subunit, particularly the N-terminal one-third and the C-terminal regions as is true of other homologs in fungi (Fig. 2B). Subsequently, a PSI-BLAST search was performed using the human B-block binding subunit as a query of the fungi database. Although the *S. cerevisiae *B-block binding subunit was hit with an *E*-value of 5e^-7^, the *Ustilago *sequence of GI 46100591 was not hit with an *E*-value better than 10 (data not shown). Moreover, a PSI-BLAST search performed using the *Arabidopsis *homolog (GI 25402830) as a query of the fungi database also did not hit the *Ustilago *sequence of GI 46100591 with an *E*-value better than 10, although the *S. cerevisiae *and *S. pombe *B-block binding subunits were hit with *E*-values of 0.93 and 9.7 respectively (data not shown). These results indicate that animal and plant B-block binding subunits are more similar to the yeast subunits than to the *Ustilago *protein GI 46100591. The overall results in this section demonstrate that the greater similarity between the plant and animal B-block binding proteins extends to the green alga protein, and the greater differences in fungi go beyond the yeast taxa.

## Discussion

In this study, I have demonstrated that the B-block binding subunits of TFIIICs in vertebrates are apparently homologous to those of yeasts, by identifying the homologs of each in *Arabidopsis*. The *Arabidopsis *proteins (GIs 25402830, 9665127, 15218016, and 25404859), which show strong similarity to B-block binding subunits, are the hypothetical proteins translated conceptually from the nucleotide sequences of the chromosome I [[30]; see also ]. The lengths of the inferred amino acid sequences of three of these *Arabidopsis *proteins (GIs 25402830, 9665127, and 15218016), are close to those of the amino acid sequences of the vertebrate subunits (Fig. 2). These *Arabidopsis *proteins probably function as the B-block binding subunits *in vivo*. B-block binding subunits act on the RNAP III promoters, the sequences of which are conserved in diverse eukaryotes (Background; Fig. 1). Thus, the domains of the subunits that bind to these promoters also should be conserved in vertebrates and yeasts. The N-terminal one-third regions of the human, yeast, and the *Arabidopsis *homologs found in this study probably associate with the B-block sequences. It is interesting that the HMG boxes predicted in the *S. cerevisiae *B-block binding subunit [14] overlap with the regions conserved in many of these putative B-block binding subunits.

There is a striking degree of similarity in most of the RNAP III transcription machinery in human, *S. pombe*, and *S. cerevisiae*; RNAP III, TFIIIA, TFIIIB, and the TFIIIC subunits that interact with the transcription initiation site, are highly conserved in these three organisms [31]. On the other hand, the TFIIIC subunits, which interact with downstream promoter regions including the B-block binding subunits, are more divergent [31]. There is the possibility that substitution rates of the amino acid residues in the B-block binding subunits vary among animals, fungi, and plants, resulting in the high divergence between the human and fungi proteins, and the similarity between the human and plant proteins. Alternatively, evolutionary inferences based on the RNAP III transcription machinery may be different from those of the genes that generally have been used to examine phylogenetic relationships in animals, fungi, and plants. RNAP III transcribes genes encoding tRNA, 5S rRNA, and several viral RNAs, and SINEs (Background). It was reported that molecular phylogenies based on tRNA sequences place plants as the sister group to the animals, although the tRNA data set available at the time was small [32]. Generally, it is thought that 5S rRNA is convenient for intrakingdom phylogenies, but cannot resolve the question of the animal-plant-fungal divergence because of its short length and high divergence [32,33]. It should be noted that more recent investigations of the proteins involved in RNA metabolism, the mRNA capping apparatus, and several key components that regulate the cell cycle, also suggest a close relationship between animals and plants, with fungi as more distant [34-36].

## Conclusions

Previously, no evidence of homology between the B-block binding subunits of TFIIICs of vertebrates and yeasts has been reported. PSI-BLAST searches presented here provided the evidence that these subunits are homologous, and that the *Arabidopsis *proteins can be used to link them. These results imply that, with respect to the B-block binding subunits, animals are evolutionarily closer to *Arabidopsis *than to yeasts. Comparisons of the B-block binding proteins from additional plant taxa showed that the greater similarity between plants and animals extends to the green algae *Chlamydomonas*. It was also demonstrated that the differences in fungi go beyond the yeast texa, and occur in basidiomycetes. These are interesting because molecular phylogenetic analyses using the small subunit rRNA and ubiquitous proteins, show that fungi are more closely related to animals than either is to plants.

## Methods

To search for similarities between the B-block binding subunits of vertebrates and yeasts, I used the PSI-BLAST program in the NCBI website [19]. PSI-BLAST searches were performed by default: matrix was BLOSUM62; gap costs were Existence 11 and Extension 1; and the *E*-value of threshold was 0.005. Peptide sequence databases used for the PSI-BLAST searches were all non-redundant GenBank CDS translations, RefSeq proteins, PDB, SwissProt, PIR, and PRF (total 1605642 sequences). The PSI-BLAST was limited searches of the eukaryota databases, when the amino acid sequences from the *Oryza *and *Chlamydomonas *coding regions were used as queries. The fungi database was used in a search for homologs of the *S. pombe *B-block binding subunit in basidiomycetes. PSI-BLAST was run three times for each of the queries. To search for homologs of the *Arabidopsis *protein GI25402830 in the plant sequences, the tblastn program was used [19]. To search for homologs of the *Arabidopsis *protein GI25402830 in the *Chlamydomonas reinhardtii *sequences, the tblastn program at the Joint Genome Institute website , was used. Clustal W in the EMBL-EBI website  was used to align the multiple amino acid sequences [21]. Clustal W was performed by default: matrix was Gonnet 250; the penalty for opening a gap was 10; the penalty for extending a gap was 0.05; and gap separation penalty was 8. Secondary structures of the proteins were predicted by using the PSIPRED protein structure prediction server (PSIPRED v2.4 in ) [29].
